# Effect of the new 75-mg orodispersible film of sildenafil on erection and sexual quality of life: insights from an observational study

**DOI:** 10.1093/sexmed/qfac007

**Published:** 2023-03-01

**Authors:** Andrea Sansone, Valeria Frangione, Arturo Lanzarotti, Andrea Cocci, Carlo Ceruti, Marco De Sio, Ciro Imbimbo, Vincenzo Mirone, Luigi Schips, Carlo Terrone, Emmanuele A Jannini

**Affiliations:** Section of Endocrinology and Medical Sexology (ENDOSEX), University of Rome Tor Vergata, Rome, Italy; IBSA Institut Biochimique SA, Pambio-Noranco, Switzerland; IBSA Institut Biochimique SA, Pambio-Noranco, Switzerland; Section of Urology, Careggi Hospital, University of Florence IBSA Institut Biochimique SA, Pambio-Noranco, Florence, Italy; Division of Urology, University of Turin, Turin, Italy; Urology Unit, University “Luigi Vanvitalli” of Naples, Naples, Italy; Andrology Unit, University “Federico II” of Naples, Naples, Italy; Urology Unit, University “Federico II” of Naples, Naples, Italy; Department of Urology, SS Annunziata Hospital, University of Chieti, Chieti, Italy; Department of Urology, IRCCS Ospedale Policlinico San Martino, University of Genova., Genova, Italy; Section of Endocrinology and Medical Sexology (ENDOSEX), University of Rome Tor Vergata, Rome, Italy

**Keywords:** sildenafil, orodispersible, film, 75-mg, erection, quality of life, IIEF

## Abstract

**Background:**

The newly devised orodispersible film (ODF) of sildenafil is the first phosphodiesterase type 5 inhibitor (PDE5i) available in a 75-mg dose. This intermediate dose and the particular properties of the ODF formulation can improve the clinical management of erectile dysfunction (ED) patients.

**Aim:**

We investigated the effects of the sildenafil ODF 75-mg dose on both sexual quality of life and erectile function based on the results from an observational study in daily practice in Italy.

**Methods:**

This study was a post hoc analysis of results from an observational, real-life study carried out in ED patients at 6 treatment centers in Italy. All subjects were asked to take the prescribed dose of sildenafil ODF at inclusion (visit 1) and to return for a control visit (visit 2) to confirm or adapt the prescribed dose after a minimum of 4 weeks. An end of study control visit (visit 3) was performed after additional 4 weeks.

**Outcomes:**

Erectile function, assessed by the International Index of Erectile Function–Erectile Function (IIEF-EF) domain; sexual quality of life, measured using the sexual quality of life instrument for men (SQoL-M).

**Results:**

Among the 36 subjects initially recruited for the 75-mg dose, 5 patients dropped out of the study (2 at visit 2 and 3 at visit 3), none of whom due to treatment inefficacy or serious adverse events. At visit 2, the mean (SD) IIEF-EF scores significantly increased (∆ = 7.97 [4.71], *P* < 0.0001) as SQoL-M scores also did (∆ = 10.76 [10.46], *P* < 0.0001). At visit 3, IIEF-EF and SQoL-M scores were still significantly improved compared to baseline (∆ = 10.64 [7.01], *P* < 0.0001, and ∆ = 18.15 [12.32], *P* < 0.0001, respectively). By ANCOVA, we found no significant effects for age, BMI, previous use of PDE5i, presence of metabolic comorbidities, or smoking habits on study outcomes at both visits 2 and 3.

**Clinical implication:**

The new 75-mg ODF sildenafil formulation is a safe and effective treatment for ED, significantly improving both erectile function and sexual quality of life in patients undergoing treatment.

**Strengths and limitations:**

This is the first study assessing the efficacy of the sildenafil ODF 75-mg dose in a real-life setting. However, the small sample size, possible underlying cultural factors, and limited availability of clinically relevant data may have affected the reliability of our results.

**Conclusion:**

The use of the 75 mg ODF formulation for sildenafil represents an effective and safe novel treatment option for ED patients.

## Introduction

Erectile dysfunction (ED) is not a life-threatening condition, but ED carries a significant psychosocial burden, owing to the association of erection with virility and sexual prowess.^1^^,^^2^ ED is a reliable marker of general health, and in particular of cardiovascular health.^3-5^ In fact, erection depends on the integrity of several systems, including vascular and endocrine function, and ED shares many risk factors with other chronic diseases.^6-8^ As a systemic indicator of endothelial dysfunction, ED can be a predictor of future cardiovascular events.^3^ However, due to a plethora of reasons, including poor awareness, stigmatization, and shame, men with ED (as well as those suffering from premature ejaculation) are rarely likely to seek medical consultation for their sexual health issues.^9^^,^^10^ However, a careful and thorough assessment of the relevant risk factors and comorbidities is mandatory to address ED and provide the necessary treatments. The clinical management of ED has been drastically changed by the discovery and subsequent approval of phosphodiesterase type 5 inhibitors (PDE5i).^11^ To understand the mechanism of action of these drugs, it is necessary to know the physiological mechanism of erection, based on the nitric oxide (NO)–cyclic GMP (cGMP) pathway.^12^^,^^13^ Following sexual stimulation, NO is released from nerve terminations and from endothelial cells, stimulating guanylate cyclase activity. The resulting accumulation of cGMP leads to decreased intracellular Ca^2+^ levels and smooth muscle cell relaxation, and therefore to increased blood inflow; the lacunar spaces of the corpora cavernosa thus become engorged, reducing venous outflow, and ultimately resulting in the erectile response. The PDE5 enzyme promotes the return to a flaccid state by hydrolyzing cGMP to GMP, therefore halting smooth muscle cell relaxation and shutting off pro-erectile molecular mechanisms. PDE5is act by preventing the degradation of cGMP by the PDE5 enzyme, therefore extending the duration of the physiologic molecular response to NO and thus increasing penile rigidity and improving erection.

PDE5is are highly effective, well tolerated, and have few contraindications; however, despite all of these favorable factors, most users discontinue therapy during the first year of treatment.^14^^,^^15^ Many studies have investigated the many reasons for this treatment dropout, reporting that in most cases a combination of both medical (eg, fear of side effects, inadequate response, poor compliance) and psychosocial factors (eg, treatment cost, couple issues, perception of poor intercourse spontaneity) can drive a patient toward discontinuation of therapy.^14^^,^^15^

The introduction of the new sildenafil orodispersible film (ODF) formulation on the market by IBSA has rekindled the interest of many ED patients in treatment. This ODF formulation has several unique properties that make it highly desirable for patients, including its ease of use. In addition, the ODF formulation is currently the only option that is virtually free from the risk of counterfeiting.^16^^,^^17^ As the film dissolves in the user’s mouth, drugs delivered in this way can be taken even in the absence of water^18^ and can be easily swallowed by people with dysphagia.^19^ Additionally, such films are more robust to physical deformation than standard tablets.^20^ The bioavailability of the active pharmacological ingredient, however, is not significantly different between ODF and tablet formulations,^21^^,^^22^ The ODF formulation is also the only short-acting PDE5is having an “intermediate” dose, namely 75 mg, which allows for more tailored treatment. This intermediate dose might be beneficial in managing patients who do not respond adequately to a moderate (50 mg) dose but develop side effects upon taking the maximum available dose (100 mg). The available dose options may address this gap in the medical management of ED patients; indeed, some patients might voluntarily reduce the prescribed dose in order to limit potential side effects, without considering that the same behavior could potentially reduce drug efficacy.^23^^,^^24^ Providing a “less-than-maximum” dose can potentially improve acceptance by the patient, giving him the idea that his condition is not so severe as to require the maximum available dose of the drug; at the same time, because the intermediate dose allows better titration of the drug by providing an additional dose step between 50 and 100 mg, the 75-mg ODF can be a viable starting dose for treatment-naive patients. Although it is generally accepted that starting with a low dose and progressively titrating it to the maximum tolerable dose would be in the best interest of the patient,^25-28^ the availability of an intermediate dose could change the treatment approach for newly diagnosed patients.

Despite its availability, the new dose of sildenafil ODF has not been extensively studied. In particular, factors affecting its efficacy in erectile function (EF) and sexual quality of life have never been addressed. To improve the clinical management of ED patients, it is an absolute necessity to thoroughly understand the potential reasons for treatment failure. In the present study, we aimed to investigate the effects of the 75-mg dose of sildenafil ODF on both sexual quality of life and EF based on results from an observational study performed in daily practice in Italy.

## Materials and methods

### Study design and population

This study was a post hoc analysis of results from an observational study carried out in 6 centers in Italy (each with inclusion of at least 1 patient taking a 75-mg sildenafil dose) and performed in a real-life population of men presenting with a complaint of ED and treated in accordance with the customary clinical practice of the individual investigators. Patients were considered eligible for inclusion if they were 18 years of age or older, willing to sign informed consent, and had ED according to International Consultation on Sexual Medicine criteria.^29^ Patients were excluded if they had any contraindications to PDE5i treatment (such as concomitant use of organic nitrates/nitrites, alpha-blockers, antihypertensives, or cytochrome P450 enzyme inhibitors) or previous history of hypersensitivity to sildenafil. Additionally, patients with poor cardiovascular health, for whom sexual activity would be inadvisable according to Princeton III Consensus recommendations,^30^ were similarly excluded from the study.

This observational study was designed and monitored with consideration of the ethical principles of the International Conference on Harmonisation (ICH), the Declaration of Helsinki and its amendments, and the current guidelines for observational trials. The study was registered on the European Union Electronic Register of Post-Authorisation Studies (EU PAS Register, study reference: EUPAS25496) and submitted to the reference Ethics Committee of each participating site. Due to the observational nature of the study, a control group was not included. All subjects were asked to take the prescribed dose of sildenafil ODF (Rabestrom, IBSA; the product is registered in other countries with the following brand names: Silandyl, Silvir, and Xybilun) at study entry (visit 1) and to return for a control visit (visit 2) to confirm or adapt the prescribed dose after a minimum of 4 weeks. An end-of-study control visit (visit 3) was performed after an additional 4 weeks. Patients were encouraged to attempt sexual intercourse using the drug on at least 8 occasions during the period between visits.

### Study objectives

In the present post hoc analysis, the primary objective was to measure the efficacy of the sildenafil 75-mg ODF product with regard to EF, as assessed by the EF domain of the International Index of Erectile Function (IIEF).^31^

The secondary objective was to measure the effects of the same treatment on the sexual quality of life (SQoL) of ED patients, according to the validated sexual QoL instrument for men (SQoL-M).^32^

### Statistical analysis

Assessment of normality was performed using the Shapiro–Wilk test of normality. Data were reported as mean (SD) or median (IQR) according to distribution. The Student t-test for paired data and ANCOVA for repeated measures were used in the assessment of normally distributed data. The Mann-Whitney U-test and Wilcoxon test were used in the groups which do not agree with normal distribution. All statistical analyses and data processing were performed using Statistical Analysis Software (SAS) version 9.4 or higher (SAS Institute Inc.).

## Results

Among the 36 subjects initially recruited at visit 1 for the 75-mg dose, 3 patients dropped out of the study at visit 2, and 2 dropped out at visit 3, for a total of 33 (91.7%) and 31 (86.1%) patients for visits 2 and 3, respectively. Two of these subjects voluntarily withdrew from the study, and 3 were lost to follow-up. None of the subjects withdrew due to treatment inefficacy or serious adverse events.

### Effects of treatment at visit 2

#### Erectile function

The mean IIEF-EF scores (Table 1) increased from the mean baseline at visit 2 (∆ = 7.97 [4.71], *P* < 0.0001) in the overall population. We subsequently investigated, using ANCOVA, the effects of different variables on treatment efficacy. At visit 2, the mean IIEF-EF scores were significantly higher than at baseline in subjects aged 18–64 years (∆ = 7.86 [5.13], *P* < 0.0001) and 65–84 (∆ = 8.17 [4.06], *P* < 0.0001), with no statistically significant difference according to age (*P* = 0.84). Likewise, IIEF-EF scores increased significantly in both healthy weight (∆ = 7.08 [2.99], *P* < 0.0001) and overweight/obese patients (∆ = 8.55 [5.55], *P* < 0.0001), with no statistically significant difference between the 2 groups (*P* = 0.421). Treatment with sildenafil 75-mg ODF was effective in both naive patients (∆ = 7.58 [4.53], *P* < 0.0001) and in those with prior history of PDE5i use (∆ = 8.85 [5.1], *P* < 0.0001) without any statistically significant difference (*P* = 0.584). Similarly, no statistically significant difference was found in association with symptom duration (*P* = 0.597), with good efficacy reported in patients with an ED history of less than 2 years (∆ = 8.25 [4.97], *P* < 0.0001) or more than 2 years (∆ = 7.71 [4.58], *P* < 0.0001). The presence of diabetes and hypercholesterolemia did not affect the outcomes of treatment (*P* = 0.378), which was effective in healthy individuals (∆ = 8.21 [5.19], *P* < 0.0001) and in patients with metabolic comorbidities (∆ = 7.33 [3.24], *P* = 0.0001). Erectile function improved both in smokers (∆ = 7.29 [3.67], *P* < 0.0001) and nonsmokers (∆ = 8.47 [5.39], *P* < 0.0001), and treatment was equally effective in both groups (*P* = 0.885).

**Table 1:** IIEF-EF scores for the 75-mg sildenafil ODF study population.

	**No. of subjects**	**IEF-EF score, mean (SD)** ^a^		
		**Baseline (visit 1)**	**Visit 2**	**Visit 3**
**Overall**	33	14.27 (5.60)	22.24 (5.96)^*^	24.91 (5.36)^*^
**Age, years**				
18-64	21	14.24 (5.55)	22.10 (5.78)^*^	25.48 (4.48)^*^
65-84	12	14.33 (5.93)	22.50 (6.53)^*^	23.92 (6.75)^*^
**BMI**				
20-24	13	14.62 (5.97)	21.69 (6.84)^*^	25.08 (6.61)^*^
25-37	20	14.05 (5.49)	22.6 (5.48)^*^	24.8 (4.56)^*^
**History of PDE5i use**				
No	19	14.68 (5.89)	22.23 (5.82)^*^	25.0 (5.58)^*^
Yes	13	13.38 (5.41)	22.23 (6.64)^*^	25.0 (5.42)^*^
**Symptom duration, years**				
<2 years	16	14.81 (5.62)	23.06 (5.13)^*^	26.56 (2.73)^*^
≥2 years	17	13.76 (5.7)	21.47 (6.72)^*^	23.35 (6.73)^*^
**Metabolic comorbidities**				
No	24	14.92 (5.99)	23.13 (5.67)^*^	24.92 (6.14)^*^
Yes	9	12.56 (4.19)	19.89 (6.43)^*^	24.89 (2.62)^*^
**Smoking habit**				
Nonsmoker	14	16.21 (5.26)	23.5 (6.55)^*^	26.64 (2.92)^*^
Current smoker	19	12.84 (5.53)	21.32 (5.49)^*^	23.63 (6.4)^*^

Abbreviations: IEF-EF, International Index of Erectile Function–Erectile Function domain; ODF, orodispersible film; PDE5i, phosphodiesterase type 5 inhibitor.

^a^  ^*^P < .001 compared to baseline.

#### Sexual quality of life

Treatment was also highly effective for SQoL (Table 2), and effectiveness had increased from baseline at visit 2 (∆ = 10.76 [10.46], *P* < 0.0001) in the overall population. We performed ANCOVA on SQoL-M scores as well, looking for variables that may have influenced treatment outcomes. A significant improvement in SQoL was found in men aged 18–64 years (∆ = 10.81 [10.31], *P* = 0.0001) and older than 65 years (∆ = 10.67 [11.19], *P* = 0.007), with no significant difference (*P* = 0.779). SQoL was also increased following treatment in both normal weight (∆ = 9.54 [10.27], *P* = 0.0058) and overweight/obese patients (∆ = 11.55 [10.77], *P* = 0.0001), without any significant effect of BMI on SQoL-M scores (*P* = 0.521). Patients naive to treatment (∆ = 11.42 [9.28], *P* < 0.0001) and those with prior use of PDE5i (∆ = 10.62 [12.35], *P* = 0.009) had similar improvements for SQoL (*P* = 0.961). Longer history of ED symptoms was not associated with different outcomes for SQoL following treatment (*P* = 0.7904), with a significant improvement both in patients with less than 2 years of ED (∆ = 10.38 [8.82], *P* = 0.0003) and those with longer ED durations (∆ = 11.12 [12.07], *P* = 0.0016). Patients with diabetes and hypercholesterolemia had significantly increased SQoL-M scores (∆ = 12.22 [9.0], *P* = 0.0036), as well as those without the same conditions (∆ = 10.21 [11.09], *P* = 0.0002), with no significant difference (*P* = 0.635); likewise, smokers (∆ = 11.21 [9.44], *P* < 0.0001) and nonsmokers (∆ = 10.14 [12.05], *P* = 0.0077) had similar improvements in SQoL (*P* = 0.759).

**Table 2:** SQoL-M scores for the 75-mg sildenafil ODF study population.

	**No. of subjects**	SQoL-M **score, mean (SD)**^a^		
		**Baseline (visit 1)**	**Visit 2**	**Visit 3**
**Overall**	33	32.70 (12.36)	43.45 (13.60)^*^	50.85 (11.87)^*^
**Age, years**				
18-64	21	31.0 (12.78)	41.81 (14.85)^*^	49.33 (13.11)^*^
65-84	12	35.67 (11.48)	46.33 (11.1)^*^	53.5 (9.23)^*^
**BMI**				
20-24	13	31.85 (13.15)	41.38 (14.26)^*^	48.77 (11.39)^*^
25-37	20	33.25 (12.13)	44.8 (13.35)^*^	52.2 (12.26)^*^
**History of PDE5i use**				
No	19	30.95 (11.19)	42.37 (12.4)^*^	48.89 (10.35)^*^
Yes	13	33.69 (13.38)	44.31 (15.89)^*^	53.54 (14.16)^*^
**Symptom duration**				
<2 years	16	32.25 (11.75)	42.63 (13.24)^*^	51.88 (10.87)^*^
≥2 years	17	33.12 (13.25)	44.24 (14.29)^*^	49.88 (12.99)^*^
**Metabolic comorbidities**				
No	24	32.79 (12.5)	43.0 (13.59)^*^	48.96 (12.17)^*^
Yes	9	32.44 (12.71)	44.67 (14.37)^*^	55.89 (9.89)^*^
**Smoking habit**				
Nonsmoker	14	32.57 (13.18	42.71 (10.37)^*^	49.36 (9.13)^*^
Current smoker	19	32.79 (12.08	44.0 (15.83)^*^	51.95 (13.68)^*^

Abbreviations: SQoL-M, Sexual Quality of Life instrument for Men; ODF, orodispersible film; PDE5i, phosphodiesterase type 5 inhibitor.

^a^  ^*^P < .001 compared to baseline

### Effects of treatment at visit 3

#### Erectile function

**Figure 1:**
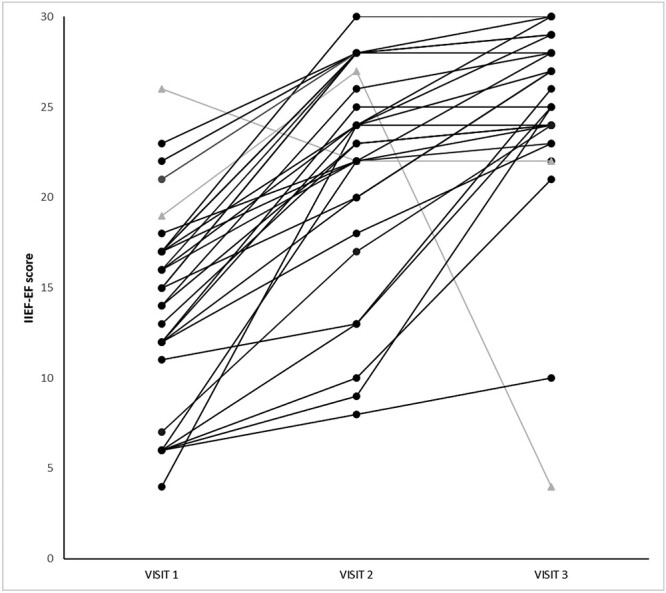
Individual IIEF-EF scores for the sildenafil ODF 75 mg study population (*n* = 33). Treatment responders (*n* = 31) are shown as black lines and points; nonresponders (*n* = 2) shown as grey lines and points. Abbreviations: IEF-EF, International Index of Erectile Function–Erectile Function domain; ODF, orodispersible film.

The same analysis was also performed at visit 3 to provide additional insight on the long-term efficacy of the 75-mg sildenafil intermediate dose (Table 1). Compared to baseline, the mean IIEF-EF scores increased in the overall population (∆ = 10.64 [7.01], *P* < 0.0001). Overall, only 2 patients among the study participants (2/33, 6.06%) failed to achieve a significant improvement while undergoing treatment (Figure 1). As previously, we used ANCOVA to investigate the effects of different variables on response to treatment. The mean IIEF-EF scores significantly increased in the group of patients aged 18–64 years (∆ = 11.24 [5.83], *P* < 0.0001) and those aged 65–84 years (∆ = 9.58 [8.91], *P* < 0.0001), with no significant effects of age on the outcome (*P* = 0.425). Both healthy weight (∆ = 10.46 [8.91], *P* = 0.0012) and overweight/obese patients (∆ = 10.75 [5.71], *P* < 0.0001) had significant improvements compared to baseline, with no statistically significant difference between the 2 groups (*P* = 0.927). Both patients naive to PDE5i (∆ = 10.32 [8.37], *P* < 0.0001) and those who previously used such drugs (∆ = 11.62 [4.59], *P* < 0.0001) reported a significant increase in IIEF-EF scores, but as for visit 2, no statistically significant difference was found between the 2 groups (*P* = 0.902). In regard to symptom duration, treatment improved EF both in patients with recent-onset ED (∆ = 11.75 [5.65], *P* < 0.0001) and in those with a longer history of disease (∆ = 9.59 [8.12], *P* = 0.0002), without any significant difference (*P* = 0.104). Treatment with sildenafil 75-mg ODF was equally effective (*P* = 0.855) in restoring EF of subjects with (∆ = 12.33 [3.5], *P* < 0.0001) or without (∆ = 10.0 [7.91], *P* < 0.0001) diabetes and hypercholesterolemia, and likewise no difference was found (*P* = 0.182) for IIEF-EF score change between smokers (∆ = 10.79 [8.36], *P* < 0.0001) and nonsmokers (∆ = 10.43 [4.93], *P* < 0.0001).

#### Sexual quality of life

The effects of treatment on SQoL were still present at visit 3 (∆ = 18.15 [12.32], *P* < 0.0001) in the overall population (Table 2). As a follow-up to our previous analysis, we also performed ANCOVA on SQoL-M scores for visit 3. Both age groups reported a beneficial effect of treatment, with no significant difference (*P* = 0.602) between patients aged 18–64 years (∆ = 18.33 [12.52], *P* < 0.0001) and those aged 65–84 years (∆ = 17.83 [12.52], *P* = 0.0004). Similarly, the effect of BMI was nonsignificant (*P* = 0.4682), with significant improvements in SQoL for both normal weight (∆ = 16.92 [10.59], *P* < 0.0001) and overweight/obese patients (∆ = 18.95 [13.54], *P* < 0.0001). SQoL improved significantly both in previous users of PDE5i (∆ = 19.85 [14.93], *P* = 0.0004) and naive patients (∆ = 17.95 [10.05], *P* < 0.0001), with no significant difference (*P* = 0.3982). In agreement with findings at visit 2, patients with shorter symptom duration (∆ = 19.63 [10.95], *P* < 0.0001) and those with duration longer than 2 years (∆ = 16.76 [13.68], *P* = 0.0001) experienced better outcomes for SQoL, without any statistically significant difference between groups (*P* = 0.523). SQoL-M increased significantly from baseline to visit 3 both in patients with (∆ = 23.44 [6.37], *P* < 0.0001) and those without (∆ = 16.17 [13.5], *P* < 0.0001) metabolic comorbidities, but once again no significant difference between groups was observed (*P* = 0.08). The same findings applied with regard to smoking, with both smokers (∆ = 19.16 [10.47], *P* < 0.0001) and nonsmokers (∆ = 16.79 [14.78], *P* = 0.0009) reporting a significant increase in SQoL-M scores but without any difference between groups (*P* = 0.512).

### Changes between visits 2 and 3

In order to retrieve further information on the long-term efficacy of the sildenafil 75-mg ODF formulation in both visits, we also investigated whether the change in ED occurring between visits 2 and 3 reached statistical significance. Both IIEF-EF (∆ = 2.67 [6.33], *P* = 0.0214) and SQoL-M (∆ = 7.39 [9.33], *P* < 0.0001) improved significantly between the 2 visits, suggesting that beneficial effects obtained from the treatment increase with prolonged use.

### Safety

The most commonly reported treatment-related adverse events (Table 3) were headache, reported by 10 patients (30.3%) followed by flushing and ocular hyperemia (5, 15.2%), hyperhidrosis (3, 9.1%), and back pain (2, 6.1%). The reported data are consistent with the known safety profile of the product, although the prevalence of headaches was slightly higher than expected, probably due to the small sample size. None of the adverse events was serious, and none led to study discontinuation or dose change.

**Table 3:** Summary of treatment-related adverse events following use of the 75-mg sildenafil ODF formulation

		**No. of events**	**No. of subjects**	**Subjects, % (*n* = 33)**
**Eye disorders**				
	Total	5	5	15.2%
	Ocular hyperemia	5	5	15.2%
**Gastrointestinal disorders**				
	Total	1	1	3.0%
	Nausea	1	1	3.0%
**General disorders and administration site conditions**				
	Total	1	1	3.0%
	Fatigue	1	1	3.0%
**Musculoskeletal and connective tissue disorders**				
	Total	4	3	9.1%
	Arthralgia	1	1	3.0%
	Back pain	3	2	6.1%
**Nervous system disorders**				
	Total	14	10	30.3%
	Dizziness	1	1	3.0%
	Headache	13	10	30.3%
**Psychiatric disorder**				
	Total	1	1	3.0%
	Insomnia	1	1	3.0%
**Reproductive system and breast disorders**				
	Total	1	1	3.0%
	Ejaculation disorder	1	1	3.0%
**Respiratory, thoracic, and mediastinal disorders**				
	Total	1	1	3.0%
	Nasal congestion	1	1	3.0%
**Skin and subcutaneous tissue disorders**				
	Total	3	3	9.1%
	Hyperhidrosis	3	3	9.1%
**Vascular disorders**				
	Total	7	6	18.2%
	Flushing	6	5	15.2%
	Hot flush	1	1	3.0%

Abbreviation: ODF, orodispersible film.

## Discussion

The present study was aimed at investigating the efficacy of the sildenafil 75-mg ODF formulation in a real-life setting, in terms of both EF and SQoL. PDE5is are available, in Europe and in several countries, in low doses (avanafil 50 mg, sildenafil 25 mg, tadalafil 5 mg, vardenafil 5 mg), in starting doses (100, 50, 10, and 10 mg, respectively), and in maximal doses (200, 100, 20, and 20 mg, respectively).^33^ The new intermediate 75-mg dose of sildenafil, coupled with the “intimacy-sparing” pharmaceutical ODF form,^16^ has raised new interest in the andrological community. Previous evidence suggested, in fact, that patients satisfaction with the traditional doses and pharmaceutical forms of PDE5is was not complete.^9^^,^^10^ We demonstrated that for both study outcomes, the intermediate dose was highly effective. A mean increase of the IIEF-EF score of 7.97 (4.71) is suggestive of a clinical benefit for patients undergoing this treatment after just 4 weeks; similarly, and interestingly, subjects who kept using the drug until the end of the study showed no loss of efficacy, with a mean increase of the IIEF-EF score of 10.64 (7.01) compared to baseline. Individual IIEF-EF scores showed that only 2 patients had an inadequate response to treatment (Figure 1), despite the positive effects reported in the overall population.

It is worth noting that none of the patients who dropped out of the study (voluntarily or lost to follow-up) reported inadequate response to treatment, possibly suggesting that the new dose could be considered a viable starting dose, at least in particular subsets of patients. We also investigated whether different factors could influence treatment response. In fact, because inadequate response to PDE5i is among the main causes of treatment discontinuation,^15^ addressing potential issues with drug dosing could improve patient compliance. In our study population, we found that treatment was just as effective across all study groups, excluding the 2 nonresponders, independently of age, BMI, history of previous use of PDE5i, presence of metabolic comorbidities, and smoking habits. This finding confirms and extends recent evidences showing that sildenafil ODF is beneficial for patients irrespective of the severity of the ED.^24^ This finding is highly relevant, as solid evidence suggests that most of these factors can affect the physiological erectile response to external stimuli. Erection is, at its core, a “hydraulic” phenomenon in which sexual stimuli, through a complex pathway involving neurological, endocrinological, and metabolic circuitry, produce a vascular response resulting in blood engorgement of the *corpora cavernosa* and, subsequently, increasing penile tumescence. Several risk factors can influence vascular dynamics, therefore impairing EF: these include endocrine disorders, such as hyperprolactinemia and hypogonadism,^34-36^ smoking,^37^ and metabolic conditions such as metabolic syndrome, diabetes, and hyperhomocysteinemia.^38^^,^^39^ Such metabolic conditions and male hypogonadism have a bidirectional pathogenic link^40^ that can be targeted by acting on lifestyles,^41^ because the benefits of physical activity, healthy nutrition, and smoking cessation act synergistically. Additionally, as available treatments are well known to the lay public,^42^ it is not uncommon for patients complaining of ED to spontaneously look online for alternative ways to acquire these drugs—in many cases, unknowingly exposing themselves to counterfeit products bearing additional risks for their health.^16^ Sexual health, on the other hand, can be the pivot of leverage to improve patient lifestyles; as such, having a drug that is highly effective, not invasive, and with quick action can potentially increase interest in health-seeking behaviors.

On top of the effects on EF, the sildenafil 75 mg ODF formulation was found to significantly improve sexual quality of life of treated men. This is in agreement with the changes in EF; however, as the SQoL-M also addresses the sense of shame, anger, guilt, and frustration in the patient, the increased scores found at visits 2 and 3 are suggestive of a better overall psychological status. As psychological and sexual health share a bidirectional relationship,^43^ this finding is also extremely important for patient care and for long-term stability of couples. Interestingly, the benefits of the sildenafil 75-mg ODF dose for SQoL do not wane over time, thus showing that the improvement is not transient or limited to the first weeks of treatment, but rather more stable and reliable over time. In a recent revision to the process of care model for management of ED it has been stated that “the treatment goals should be individualized to restore sexual satisfaction to the patient and/or couple and improve quality of life based on the patient’s expressed needs and desires.”^44^ Although preliminary, the data here suggest that sildenafil 75-mg ODF could be a strong and important new instrument to fulfill these aims.

In our study, we looked at several factors known to affect sexual health, investigating whether each of them could potentially impair response to treatment over a 4- or 8-week treatment course. We therefore performed analysis aimed to measure the change in IIEF-EF and SQoL-M scores at different times, according to age, BMI, smoking status, metabolic comorbidities, and symptom. duration. We also investigated whether prior experience with other PDE5i could influence response to the 75-mg sildenafil ODF formulation. We found that none of these risk factors have resulted in significantly different outcomes for treatment efficacy, suggesting that the treatment with this intermediate dose is equally viable for all patients on average.

Undoubtedly, the best-case scenario to investigate the efficacy of the 75-mg sildenafil ODF formulation would involve a very well-selected group of ED patients who failed to respond to a “starting dose” with a 50-mg ODF formulation, in order to assess how many patients could be “rescued” by switching to an intermediate dose before prescribing the maximum available dose. Such investigations, however, could not be performed in the present study; future research aimed in this direction could possibly help identify the best candidates for a tailored treatment with 75-mg sildenafil ODF formulations. Overall, however, the present study highlights the use of the 75-mg ODF formulation for sildenafil as a novel opportunity to take care of ED patients. Indeed, several of the benefits of the ODF formulation—such as its ease of use and rapid onset of action^13^—can make this treatment more appealing to patients than “older” formulations.

## Study strengths and limitations

This is, to our best knowledge, the first study directly investigating the effects of the sildenafil 75 mg ODF formulation on EF and sexual quality of life. This study was performed in a real-life setting, in a study population with broad inclusion criteria treated according to normal clinical practice. However, the present study also has some limitations. The small sample size, largely due to the onset of the COVID-19 pandemic, which slowed outpatient clinic activity, can affect the reliability of our results. As visits 2 and 3 were after 4 and 8 weeks of treatment, respectively, we documented that response to medication was maintained throughout the treatment, but at the same time, we could not predict treatment failure or success rates over longer periods of follow-up. For patients who were not naive to PDE5i treatment, no further analysis was made to measure whether failure to respond to previous treatments was associated with different outcomes while using the sildenafil 75-mg ODF formulation. Although no difference was found between previous users of PDE5i and patients naive to such treatment, we cannot at present provide a definite answer to whether patients who failed to respond to 100 mg sildenafil tablets would respond to the 75-mg ODF formulation. Last but not least, as hormonal^45^^,^^46^ and vascular parameters (as measured by penile duplex ultrasound^47^) were not available, we could not investigate whether treatment efficacy was different between hypogonadal and eugonadal patients, or between patients with normal and those with impaired penile hemodynamics.

## Funding

IBSA Institut Biochimique SA (Lugano, Switzerland) provided financial support for the study and for the publication of this work. A.S. and E.A.J. have also been partially supported for this work by the Italian Ministry of University PRIN (grant # 2017S9KTNE_002).

*Conflicts of interest*: E.A.J. is/has been a consultant and/or paid speaker for Bayer, IBSA, Menarini, Merck-Serono, Otsuka, Pfizer Inc, Shionogi, and viatris. V.F. and A.L. are full-time employees of IBSA. has no conflicts of interest. C.C. has been a consultant and/or paid speaker for Dompè Primary, Menarini, Takeda, Alphremev, and SOFAR. M.D.S. is/has been a paid speaker for Janssen, Astellas, and Leonardo Medica. V.M. has been a consultant and/or paid speaker for SR, Recordati, Janssen. A.C., C.I., L.S., and C.T. have no conflicts of interest to declare.
